# Hereditary Insanity

**Published:** 1848-06

**Authors:** 


*Hereditary Insanity.*—There is nothing in connexion with the study of insanity more deserving of attention than the tendency of this disease to be transmitted from parents to their offspring. The fact is most unquestionable, and we are of opinion that it has more influence in producing that disease than all other causes combined. It does not of itself excite the disease, but when it strongly exists a trivial cause will develope it. Thus most of the supposed exciting causes would of themselves be inoperative, if there was not inherited a constitutional tendency to insanity. Sometimes the children of an insane parent are, however, exempt from the disease, while it appears in the grandchildren. Contrary to the opinion of many, we have found this inherited form of insanity as curable as any other, though the subjects of it are very liable to relapse, and from slight and various causes. Sometimes a little sickness, a slight fever, or severe cold, and at others a little mental disturbance, such as the loss of relatives, or property, or religious anxiety, excite it. We have known individuals thus predisposed to insanity, have repeated attacks, and each time from a different exciting cause.—*From Dr. Winslow's Journal of Psychological Medicine.*

